# Response rate of fibrosarcoma cells to cytotoxic drugs on the expression level correlates to the therapeutic response rate of fibrosarcomas and is mediated by regulation of apoptotic pathways

**DOI:** 10.1186/1471-2407-5-74

**Published:** 2005-07-07

**Authors:** Marcus Lehnhardt, Ludger Klein-Hitpass, Cornelius Kuhnen, Heinz Herbert Homann, Adrien Daigeler, Hans Ulrich Steinau, Sonja Roehrs, Laura Schnoor, Lars Steinstraesser, Oliver Mueller

**Affiliations:** 1 Department of Plastic Surgery, Burn Center, Hand surgery, Sarcoma Reference Center, BG University Hospital Bergmannsheil, Ruhr University Bochum, Bürkle de la Camp Platz 1, 44789 Bochum, Germany; 2 Institute of Cell Biology (Tumor Research), IFZ, University of Essen, Virchowstr. 173, 45122 Essen, Germany; 3 Institute of Pathology, BG University Hospital Bergmannsheil, Ruhr University Bochum, Bürkle de la Camp Platz 1, 44789 Bochum, Germany; 4 Tumor Genetics Group, Max-Planck-Institut für molekulare Physiologie, Otto Hahnstr. 11, 44227 Dortmund, Germany

## Abstract

**Background:**

Because of the high resistance rate of fibrosarcomas against cytotoxic agents clinical chemotherapy of these tumors is not established. A better understanding of the diverse modes of tumor cell death following cytotoxic therapies will provide a molecular basis for new chemotherapeutic strategies. In this study we elucidated the response of a fibrosarcoma cell line to clinically used cytostatic agents on the level of gene expression.

**Methods:**

HT1080 fibrosarcoma cells were exposed to the chemotherapeutic agents doxorubicin, actinomycin D or vincristine. Total RNA was isolated and the gene expression patterns were analyzed by microarray analysis. Expression levels for 46 selected candidate genes were validated by quantitative real-time PCR.

**Results:**

The analysis of the microarray data resulted in 3.309 (actinomycin D), 1.019 (doxorubicin) and 134 (vincristine) probesets that showed significant expression changes. For the RNA synthesis blocker actinomycin D, 99.4% of all differentially expressed probesets were under-represented. In comparison, probesets down-regulated by doxorubicin comprised only 37.4% of all genes effected by this agent. Closer analysis of the differentially regulated genes revealed that doxorubicin induced cell death of HT1080 fibrosarcoma cells mainly by regulating the abundance of factors mediating the mitochondrial (intrinsic) apoptosis pathway. Furthermore doxorubicin influences other pathways and crosstalk to other pathways (including to the death receptor pathway) at multiple levels. We found increased levels of cytochrome c, APAF-1 and members of the STAT-family (STAT1, STAT3), while Bcl-2 expression was decreased. Caspase-1, -3, -6, -8, and -9 were increased indicating that these proteases are key factors in the execution of doxorubicin mediated apoptosis.

**Conclusion:**

This study demonstrates that chemotherapy regulates the expression of apoptosis-related factors in fibrosarcoma cells. The number and the specific pattern of the genes depend on the used cytotoxic drug. The response rates on the gene expression level, i.e. the number of genes regulated by the drugs actinomycin D, doxorubicin and vincristine, correlate to the clinical effectiveness of the drugs. Doxorubicin seems to exert its cytotoxic mechanism by regulating genes, which are involved in several different apoptosis regulating pathways. The exact knowledge of the genes affected by the drugs will help to understand the diverse modes of soft tissue sarcoma cell death in response to cytotoxic therapies.

## Background

With only 1% of all solid malignancies and more than 50 sub entities soft tissue sarcomas are rare and heterogeneous [1,2]. Adequate complete surgical resection in combination with radiotherapy is the mainstay of therapy resulting in a 50% to 80% 5-year survival rate. Recent chemotherapy studies revealed a high fraction of resistant soft tissue sarcoma. Response rates above 15% [3-5] within all variant histological subtypes were reported only for doxorubicin (adriamycin), actinomycin D and ifosfamide [6,7].

Generally, two main classes of drug resistance can be distinguished. Tumor cells are either primarily resistant to chemotherapeutic drugs (intrinsic resistance), or some of the cells respond to chemotherapy at the first treatment but the remaining cells recur later to form a multidrug-resistant tumor (acquired resistance) [8]. A single mechanism or a distinct pathway cannot explain the effectiveness of a cancer drug. In carcinomas multiple mechanisms of drug resistance have been characterized on the molecular level [9,10]. These include the overexpression of the genes p53 [11-14], MDR1 (multidrug resistance gene 1) [14-16], MRP1 (multidrug resistance-associated protein), or the induction of DNA repair [14]. In addition various regulatory genes targeted for genetic alterations during tumorigenesis may also influence cellular sensitivity to chemotherapeutic drugs. These genetic alterations involve tumor suppressor genes, oncogenes, cell cycle regulators, transcription factors, growth factor receptors, DNA repair factors and cell death regulators. Only little is known about the molecular basis of drug resistance in soft tissue sarcomas [17-20]. Comprehensive knowledge of the expression changes induced by cytostatic drugs should be useful for examining the molecular basis of drug resistance. The specific expression and response profiles of a cell line established from a distinct tumor may ultimately allow to design improved therapeutic regimes with the aim to circumvent drug resistance [20]. In this study, we used Affymetrix microarrays to monitor mRNA expression changes in an established fibrosarcoma cell line. The cells were treated with the two widely used cytotoxic drugs doxorubicin and actinomycin D. In a parallel analysis, cells were treated with vincristine as a cytostatic drug of very low response in human soft tissue sarcomas for comparison.

## Methods

### Cell Culture and RNA-preparation

HT-1080 (human fibrosarcoma cells, cell line CCl 121 from ATCC) were cultured in modified Eagle's medium supplemented with 10% FCS in 15 cm Petri dishes. Semi-confluent cultures were treated with 0,5 μg/ml doxorubicin for 6 h or 24 h, 0.1 μg/ml actinomycin D or 0.4 μg/ml vincristine for 24 h. Total RNA was purified from the cells using Trizol reagent (Life Technologies), as specified by the manufacturer. RNA integrity was assessed using the Agilent 2100 Bioanalyzer (Agilent Technologies).

### Oligonucleotide microarray analysis

For microarray analyses we used the Affymetrix Gene Chip platform employing a standard protocol for sample preparation and microarray hybridization that has been described in detail previously [20,21]. Briefly, total RNA was converted into double-stranded cDNA using an oligo-deoxythymidine primer containing the T7 RNA polymerase binding site (5'- GCATTAGCGGCCGCGAAATTAATACGACTCACTATAGGGAGA – (dT)_21_V-3') (MWG Biotech) for first strand synthesis. After generation of double-stranded cDNA from the first-strand cDNA, biotinylated cRNA was synthesized by *in vitro *transcription using the BioArray High Yield RNA Transcript Labeling Kit (Enzo Diagnostics). Labeled cRNA was purified on RNeasy columns (Qiagen), fragmented and hybridized to HG-U133A microarrays (Affymetrix). The arrays were washed and stained according to the manufacturer's recommendation and finally scanned in a GeneArray scanner 2500 (Agilent).

Array images were processed to determine signals and detection calls (Present, Absent, Marginal) for each probeset using the Affymetrix Microarray Suite 5.0 software (MAS 5.0; statistical algorithm). To determine expression changes induced by doxorubicin and vincristine, arrays were scaled across all probesets to an average intensity of 1000 to compensate for variations in the amount and quality of the cRNA samples and other experimental variables of non-biological origin. To determine target genes of actinomycin D, which induced rather strong alterations in gene expression, scaling to externally added polyA+ spike controls proved to be more approriate. Pairwise comparisons of treated *versus *control samples were carried out with MAS 5.0, which calculates the significance (change p-value) of each change in gene expression based on a Wilcoxon ranking test. To limit the number of false positives, we restricted further target identification to those probesets, which received at least one present detection call in the treated/control pair. Probesets exhibiting a signal log2 ratio >1.32 and a change p-value <0.001 or a signal log2 ratio <-1.32 and a change p-value >0.999 (corresponding to 2.5 fold up- or down-regulation) were identified by filtering using the Affymetrix Data Mining Tool 3.0 (Table 1). The normalized raw data set of this analysis can be found at the Global Environment Outlook portal at http://www.ncbi.nlm.nih.gov/geo (GEO accession code: GSE2238).

### Real-time RT-PCR and data processing

Real-time RT-PCR was performed using Taqman low density arrays on a ABI PRISM^R ^7900HT Sequence Detection System (Applied Biosystems). cDNA was generated with random primers using the High Capacity cDNA Archive Kit (Applied Biosystems) using 5 μg total RNA in a 20 μl reaction volume. Approximately 400 ng cDNA was used per port (48 wells) representing 24 different Assays-on-Demand in two replicates. Transcript levels were determined in duplicate reactions by the ΔΔCt method using 5 transcripts as an internal reference [see Additional file 1].

### GO and pathway analysis

Statistically overrepresented Gene Ontology (GO) terms within target gene lists were identified by the hypergeometric distribution implemented in the GO Browser of Spotfire DecisionSite for Functional Genomics (Spotfire). Specific signaling pathways were identified using the software package Pathway Assist™ (Stratagene).

## Results

**Figure 1:**
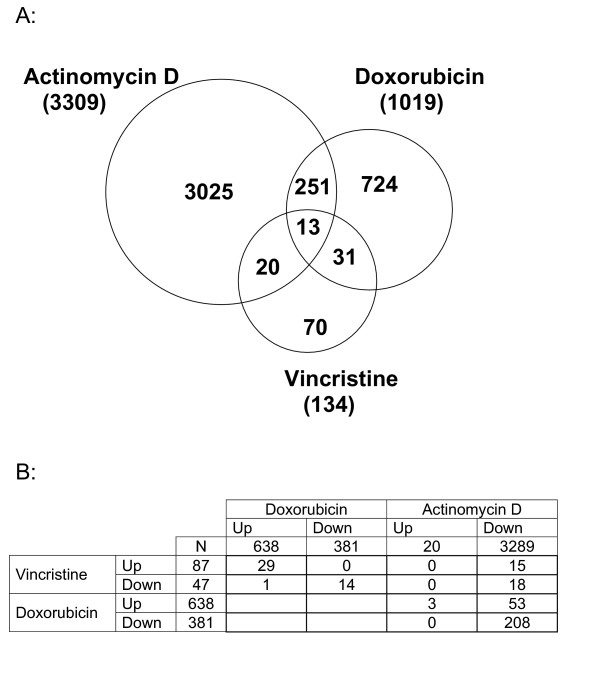
**A+B: Patterns of genes affected by Actinomycin D, Doxorubicin and Vincristin. **(A) Venn diagram showing the overlap of probesets regulated by the three different drugs in HT1080 cells after 24 h of treatment. Numbers in brackets refer to the total number of affected probesets. (B) Cross table showing the number of concordant probesets up- or down-regulated by the three different compounds.

Microarray analysis revealed the differential gene expression patterns of fibrosarcoma cells treated with cytostatic drugs. Based on the Affymetrix statistical change and detection algorithm, a stringent combination of filter criteria was used to identify significantly changed genes, while keeping the false postive rate at a minimum (Table 1; see Material and Methods for details). As indicated in Figure 1, the three different drugs proved to affect remarkably different numbers of probesets. After actinomycin D treatment for 24 hours, 3309 probesets showed differential expression levels. Of these, a vast majority of 99.4% were under-represented, confirming the general inhibitory activity of actinomycin D on RNA polymerization. In comparison, treatment with doxorubicin for 24 hours affected a lower number of probesets (1019) than actinomycin D, while 62.6 % of these probesets were up-regulated. Treatment with doxorubicin for 6 hours resulted in just 3 differentially expressed targets, indicating that a shorter incubation period leads to a minor response. Vincristine treatment for 24 hours led to the identification of 134 differentially regulated probesets. Of these, 87 probesets were increased and 47 decreased. Interestingly, the numbers of probesets, which were regulated by the different drugs, correlate with the clinical effectiveness of these three cytostatics. Vincristine, the agent with the lowest impact on gene expression has the weakest effect on the growth of fibrosarcomas in-vivo [22], while actinomycin D and especially doxorubicin appeared to have a much stronger effect on cell growth in soft tissue sarcoma [23,24].

The Venn diagram shows that there is only a limited overlap of the genes affected by the three different drugs (Figure 1). Thirteen probesets representing 12 genes were common targets of all three drugs; however, while these targets were all down-regulated by actinomycin D, doxorubicin and vincristine consistently up-regulated 4 of those transcripts. Analyzing the up- and down-regulated transcripts in more detail (Figure 1B), it is evident that 43 out of the 44 probesets common to doxorubicin and vincristine target lists are affected in the same direction (29 up, 14 down). Vincristine and actinomycin D targets lists share 33 targets, but concordant regulation was restricted to 18 down-regulated probesets. Similarly, doxorubicin and actinomycin D shared 264 common targets, while only 3 up- and 208 down-regulated transcripts were affected in the same direction. The scatter plots and moreover the dendogramm illustrate these quantitative findings (Figures 3, 4).

To get an overview of the biological processes affected by the three different drugs, we classified the regulated probesets to the various categories as defined by the gene ontology annotations (Table 2) [25]. Analyzing the distribution of the targets to the various categories, we found a significant overrepresentation in several categories that depended on the drug used.

The biochemical functions of the genes in the expression profile of the actinomycin D-treated fibrosarcoma cells are diverse and include cell proliferation, cell cycle, metabolism and others. Altered expression of the metabolism genes especially include nucleotide metabolism (Table 2, column 1). Increased expression of cell proliferation and cell cycle genes were found after doxorubicin treatment (column 2) while cells treated with vincristine significantly overexpressed metabolism (especially nucleotide metabolism) genes (column 3).

Figure 4 represents pathways analyses based on decreased and increased genes detected after 24 h treatment with doxorubicin. Because killing of cancer cells by cytotoxic therapies is predominantly mediated by triggering apotosis in target cells, we focused on differential expressed genes involved in cell death pathways.

There are two known cellular pathways, which lead to the initiation of apoptosis [9,10]. The 'extrinsic/exogenous' pathway (activated by death receptors, a subgroup of the TNF receptor super family) and the 'intrinsic/endogenous' pathway (a mitochondrial pathway controlled by members of the Bcl-2 family) [26]. Most cytotoxic drugs, which activate apoptosis, lead to the activation of the mitochondrial pathway. The expression of Bcl-2 may be responsible for resistance to apoptosis induced by cytotoxic drugs, and down-regulation of Bcl-2 sensitizes cells to doxorubicin [27]. Doxorubicin-induced cell death is mediated by the tumor suppressor p53. Remarkably, none of the drugs tested in this study changed the expression level of p53. Nevertheless, the drugs showed an influence on the expression levels of other important genes, which are involved in the intrinsic pathway (Figures 4, 5, 6). Cytochrome c expression showed an increased level, and Bcl-2 showed a decreased level after doxorubicin treatment in the fibrosarcoma cell line (Figure 4).

The apoptotic protease activating factor 1 (APAF1) was up-regulated. APAF1 plays a central role in the cell death machinery and is an activator of the caspases-cascade, especially of caspase-9. Several members of the protease family of caspases (cysteine dependent aspartate-specific proteases) play a major role in apoptosis [10]. We found up-regulated expression of caspase-1, caspase-3, caspase-6, caspase-8 and caspase-9 (Figures 4, 5, 6). Increased expression levels were found for two members of the STAT-family (signal transducer and activator of transcription): STAT1 and STAT3, too. These proteins play roles in several different apoptotic pathways (Figures 3A, D). Increased expression of STAT1 triggers tumor progression and leads to down-regulation of apoptosis [28]. STAT3 plays a key role in signal transduction and promotion of apoptosis [29]. In addition the protein is involved in interleukin signaling. Altered expression of the interleukin-family includes up-regulation of IL-6, a mediator of anti-apoptotic effects and drug-resistance mechanisms. In addition to the regulation of apoptosis activating pathways doxorubicin regulates an anti-apoptotic pathway. The insulin-like growth factor 1 receptor (IGF-1R) is an important signaling molecule in cancer cells. IGF-1R was prominently targeted for up-regulation by doxorubicin treatment.

In addition we found expression changes in genes, which are involved in the extrinsic pathway. Fas/APO-1/CD95 is a member of the tumor necrosis factor (TNF) receptor super family. We found altered expression level of different FAS family mediators. PTPN13 (increased) activates directly caspase-8. The Fas-associated factor 1 (FAF1) was down-regulated. FAS and the tumor necrosis factor (TNF) super family of receptors induce apoptosis by recruiting adaptor molecules through death domain interactions. The central adaptor molecule for these receptors is the death domain-containing protein FADD. FADD binds a death domain on a receptor or an additional adaptor and recruits caspases to the activated receptor [30,31]. FADD was up-regulated in this experiment. In contrast we found decreased expressions of FAIM and FAIM2, Fas-associated apoptosis inhibitory molecules.

Looking at non-apoptotic pathways, we found an influence of doxorubicin on the pathway of skeletal myogenesis. PCAF (p300/CBP-associated factor), a co-activator of the tumor suppressor p53, was increased. This finding is in line with the results of others [32-34]. Furthermore the transcription factor MEF2 (Myocyte enhancer factor-2), that controls critical cellular processes including proliferation, differentiation and survival, was increased.

Analyzing the proliferation activating pathways controlled by EGF and Ras we found altered expression of the GTPase activating protein Rasa-1 (Ras p21 protein activator-1). Rasa-1 is involved in the signal transduction pathway of several growth factors and in the down-regulating of different proteins of the Ras GTPase super family [34]. In addition we found the up-regulated expression of SOS1, which encodes for a Ras-specific exchange factor. Furthermore H-RAS was up-regulated, which is a member of the Ras GTPase super family.

To confirm the changes observed by microarray, we measured the expression of 46 representative apoptosis genes that were in part differently expressed in these experiments by quantitative RT-PCR using Taqman low density arrays (for details see Material and Methods). Overall the success rate of validation by RT-PCR was 62%. For securely detected probestes (pp-call) in the doxorubicin-experiment the success rate of validation was 78% [see Additional file 1].

## Discussion

Soft tissue sarcomas are very heterogeneous regarding their presentation, growth behavior and response to therapeutic interference. The presentation and behavior of these tumors depend on location and histological characteristics [24]. Standard therapy consists of complete surgical resection in combination with adjuvant radiotherapy. Because of low response rates chemotherapy is largely restricted to clinical trials. Hence, novel chemotherapy protocols that overcome resistance would be highly valuable [3].

In this study we analyzed the differential gene expression pattern of fibrosarcoma cells after treatment with the three cytostatics doxorubicin, actinomycin D and vincristine. First of all we found that the number of significantly altered probe sets directly correlates with the known clinical response rates of the three cytostatics. Treatment with vincristine shows a very low response rate in soft tissue sarcomas (134 altered probe sets). Doxorubicin (1.109 altered probe sets) and actinomycin D (3.309 altered probe sets) are more effective chemotherapeutic drugs with a significantly higher response rate in these tumors (Figure 1) [35]. This correlation suggests that a strong impact on gene expression leads to a high clinical effectiveness. Thus the microarray analysis of the differential gene expression might be a valuable tool to judge the clinical effectiveness of newly developed drug candidates.

The alkaloid vincristine inhibits mitosis by inhibiting tubulin polymerization and thus by preventing metaphase spindle formation. In addition there are hints that vincristine interferes with DNA and RNA synthesis (Figure 1). Because of response rates lower than 5% vincristine is not well established in the clinical treatment of soft tissue sarcomas [36,37]. Vincristine seems to influence the expression of several genes of different pathways rather than of distinct genes of specific pathways.

**Figure 2:**
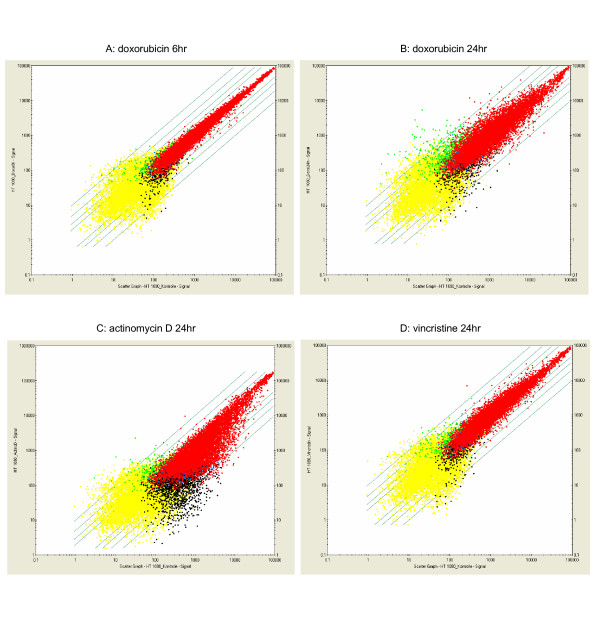
**A-D: Induction of HT1080 cells by doxorubicin, actinomycin D and vincristine. **HT1080 cells were treated with 0,5 μg/ml doxorubicin for 6 h or 24 h (A, B), 0.1 μg/ml actinomycin D (C) or 0.4 μg/ml vincristine (D) for 24 h. Expression profiles of the untreated control (x-axis) and the drug-induced (y-axis) HT1080 cells are shown as bivariate scatterplots of 14,500 genes from the microarray. The values are corrected intensities representing levels of expression for the DNA elements of the microarray. Colour legend: red: P-P = present in baseline (x-axis) and present in experiment (y-axis) black: P-A = present in baseline but absent in experiment green: A-P = absent in baseline but present in experiment yellow: A-A, A-M, M-A, M-M = absent or marginal in baseline or experiment

**Figure 3:**
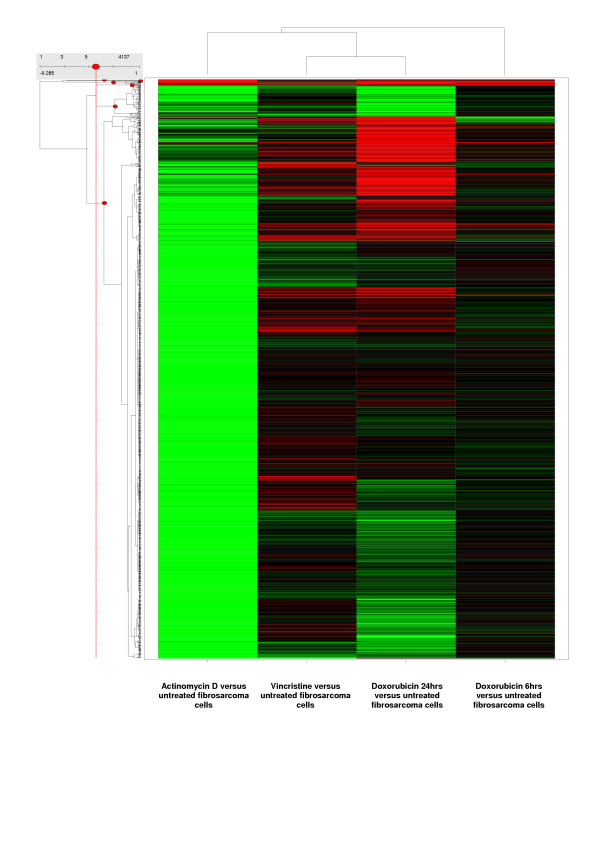
**Overall expression patterns of 4137 genes in fibrosarcoma cells treated with doxorubicin (6 h and 24 h), actinomycin D and vincristine (each 24 h). **Horizontal rows represent individual genes; vertical columns represent individual samples. Colour range: Brightest red: Signal Log Ratio (SLR)> = 2 (indicates expression level above untreated cells); brightest green: SLR< = 2 (indicates expression level below untreated cells); black: SLR = 0 (indicates unchanged expression). The dendogram at the top of the matrix indicates the degree of similarity between tumor samples; the dendogram at the left side indicates the degree of similarity among the selected genes according to their expression patterns.

With response rates between 15% and 20% actinomycin D is one of the most widely used chemotherapeutic agents in the treatment of soft tissue sarcomas [38]. Actinomycin D is known to inhibit cell proliferation in a rather non-specific way by forming a stable complex with double-stranded DNA and thus inhibiting DNA-primed RNA synthesis [39-42]. In conformance with this inhibitory effect on transcription we found 3.289 probe sets decreased and correspondingly only 20 probe sets increased in actinomycin D treated cells (Figure 1). The biochemical functions of these down-regulated genes include cell proliferation, cell cycle, programmed cell death and metabolism (Figure 2). These findings provide a first clue of the complex biological processes affected by the drug both on a quantitative and on a qualitative basis.

Doxorubicin achieves clinical response rates of 20% to 26% and represents one of the most effective chemotherapeutic agents among the available drugs, which are used in first line single drug therapies [43]. Doxorubicin inhibits topoisomerase II and causes DNA damage, at least partially in a p53-dependent manner [44]. There was a marked count of probesets affected by doxorubicin. The regulated sequences include genes involved in cell proliferation and cell cycle (Figure 2). This finding is in line with the results of others and may represent the signature profile of doxorubicin induced gene expression [8].

**Figure 4:**
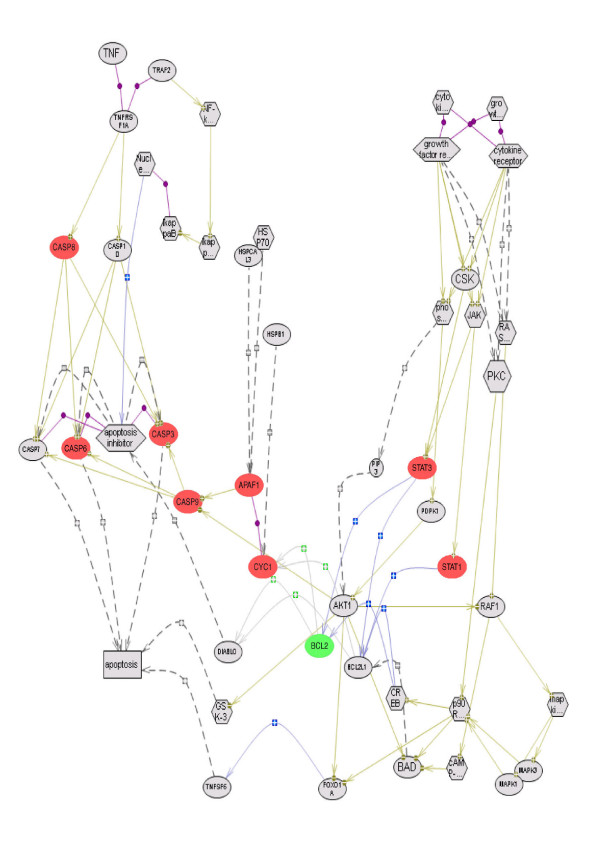
**Pathway-analyses based on decreased (green) and increased (red) genes detected in fibrosarcoma cells after 24 h treatment with doxorubicin. Results shown for apoptosis. **Signaling pathway maps were identified using the software package Pathway Assist. A minimum of 3 interacting genes is the required criteria for creation of a map (Software: Pathway Assist™ Stratagene).

Understanding the exact molecular events and the resistance mechanisms induced by anticancer therapy would provide new opportunities for pathway-based rational therapy and for drug development [9,10]. To elucidate the mechanisms and the degree of the molecular response we analyzed the intracellular pathways, which are influenced by doxorubicin in a fibrosarcoma cell line. We identified five different pathways, which include at least three genes with altered expression levels. Four of these pathways regulate apoptosis (Figure 4).

Apoptosis in response to cancer therapy proceeds through activation of the core apoptotic machinery including the receptor and the mitochondrial signaling pathway [45-47]. The relative contribution of the receptor and mitochondrial pathways to drug-induced apoptosis has been a subject of controversial discussion. While a number of initial studies suggested that cancer therapy-triggered apoptosis involves activation of the CD95 receptor/ligand system, compelling evidence subsequently indicated that the majority of cytotoxic drugs initiate cell death by triggering the mitochondrial signaling pathway [48]. The data of our study support either possibilities. Doxorubicin showed influences on genes involved in the extrinsic as well as on genes involved in the intrinsic pathway.

**Figure 5:**
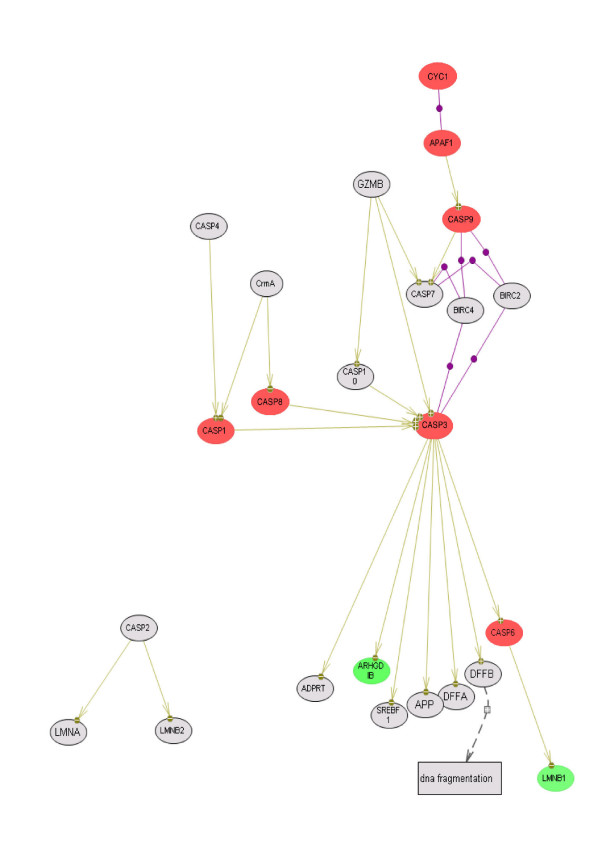
**Pathway-analyses based on decreased (green) and increased (red) genes detected in fibrosarcoma cells after 24 h treatment with doxorubicin. Results shown for caspases. **Signaling pathway maps were identified using the software package Pathway Assist. A minimum of 3 interacting genes is the required criteria for creation of a map (Software: Pathway Assist™ Stratagene).

**Figure 6:**
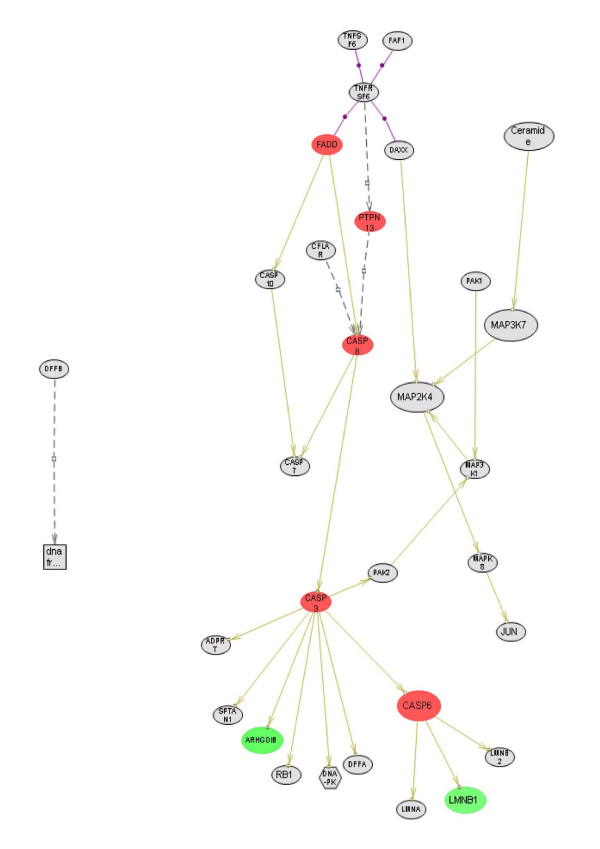
**Pathway-analyses based on decreased (green) and increased (red) genes detected in fibrosarcoma cells after 24 h treatment with doxorubicin. Results shown for FAS-signalling. **Signaling pathway maps were identified using the software package Pathway Assist. A minimum of 3 interacting genes is the required criteria for creation of a map (Software: Pathway Assist™ Stratagene).

One major extrinsic apoptogenic pathway is the cytochrome c/Apaf-1/caspase-9 dependent pathway (Figure 4). We found cytochrome c, Apaf-1, caspase-3, caspase-6, caspase-8 and caspase-9 up-regulated by doxorubicin. The release of mitochondrial apoptogenic proteins, such as cytochrome c, into the cytoplasm leads to Apaf-1 dependent activation of caspase-9, which is a key event in this pathway. Especially Apaf-1, a member of the NOD-family (nucleotide binding oligomerization domain) is known to play a central role in the cell death machinery. Its expression has been demonstrated to be involved in several cell death pathways. Furthermore Apaf-1 is an activator of caspases [49,50]. Apaf-1 inactivation leads to chemoresistance for example in malignant melanoma cells [51]. Additionally, the anti-apoptotic factor Bcl-2, a direct antagonist of p53 and cytochrome c, was down-regulated. Various pro- and anti-apoptotic Bcl-2 family proteins regulate the permeability of the mitochondrial outer membrane, and DNA damage triggers apoptosis through the down-regulation of anti-apoptotic Bcl-2. Yeh and Pusztai demonstrated that Bcl-2 could be responsible for the resistance to apoptosis induced by cytotoxic drugs. Thus the down-regulation of Bcl-2 should sensitize cells to doxorubicin [52-54]. These results might indicate that doxorubicin initiates cell death of HT1080 cells by activating the cytochrome c/Apaf-1/caspase-9 dependent pathway. Our results confirm the results of others showing that doxorubicin triggers Bcl-2 down-regulation, cytochrome c release from mitochondria and the activation of caspases-3 and 9 [55-57]. Doxorubicin changed the expression of several caspases. These findings are in agreement with recently published data about different caspases acting as key factors in doxorubicin mediated cell death [58-62]. Caspases are the key effector molecules in most forms of apoptotic cell death and represent the level, where the intrinsic and the extrinsic apoptotic pathway converge [62]. Additionally they interact with other non-caspases apoptotic pathways [63]. Caspases are categorized into initiator (caspase-2, -8, -9, -10) and effector caspases (caspase-3, -6, -7) [64]. Doxorubicin induced the expression of caspases-1, -3, -6, -8 and -9. Caspase-6 cleavage may feed back to the receptor pathway by cleaving caspase-8 [65,66]. Within the caspases pathway we found two additional genes down-regulated, LMNB1 and ARHGDIB (Figure 5). Little is known about the function of these proteins. They might play a role in a number of processes related to metastases [67,68]. LMNB1 and ARHGDIB are effectors of the caspases-3 and -6, respectively, and are thus also involved in the apoptotic Fas signaling pathway (Figure 6). There are hints that the extrinsic pathway is connected with the sensitivity and with the resistance of tumor cells towards cytotoxic treatment. Chemotherapeutic treatment led to an increase in CD95L expression, which triggered the receptor pathway. In support of this concept, increased levels of CD95L were observed in many different tumor cell lines [9,32,69-71]. Our results indicated that the pathway is influenced by doxorubicin. In addition to the expression of the two genes LMNB1 and ARHGDIB, the expression of the death domain containing protein FADD, PTPN13 (protein tyrosine phosphatase non-receptor type 13) and the expression of the caspases-8, -3 and -6 were regulated. Furthermore we found decreased expression levels of the Fas-associated apoptosis inhibitory molecules FAIM and FAIM2, what is another indication for the activation of the Fas pathway by doxorubicin.

In the intrinsic pathway, various apoptosis inducing signals directly or indirectly change mitochondria membrane permeability and cause release of mitochondrial intermembrane proteins, including cytochrome c [72]. In the cytoplasm, pro-caspase-9, Apaf-1 and cytochrome-c assemble to form the apoptosome [73,74]. In an ATP-dependent process, procaspase-9 is cleaved in the apoptosome. Activated caspase-9 then cleaves and activates down stream caspases, such as caspase-3. Down-stream of the caspase-8 or caspase-9 activation cascade the intrinsic and extrinsic apoptosis pathways converge in the executioner caspases-3, -6 and -7 [75,76]. Once activated, the executioner caspases are responsible for the proteolytic cleavage of a broad range of cellular proteins resulting in cell death [73,74].

In addition to the activation of several different apoptosis regulating pathways doxorubicin activates also pathways that activate proliferation, differentiation and inhibition of apoptosis. The EGF signaling pathway regulates cell proliferation and transformation. Analyzing this pathway we found altered expression of the genes Rasa1 (decreased levels), STAT1 and STAT3 (increased levels). STAT1 has been implicated in modulating pro- and anti-apoptotic genes following several stress-induced responses and therefore is able to act as a tumor suppressor [77]. STAT3 suppresses expression of pro-inflammatory mediators in tumor cells. Blocking STAT3 in tumor cells induces the expression of pro-inflammatory cytokines and chemokines that activate innate immunity and dendritic cells [78]. For example STAT3 protects prostatic cancer cells from apoptosis induced by T lymphocytes [79]. In human neuroblastoma cells doxorubicin leads to STAT3 activation that results in apoptosis inhibition [80].

The pleiotropic cytokine IL-6 exhibits many physiological functions, including host defense, bone metabolism and acute phase responses. A variety of malignant tumors express IL-6. In multiple myeloma cells IL-6 inhibits apoptosis induced by serum starvation, dexamethasone, and Fas [81]. In pre-B cells, however, IL-6 is important for differentiation and apoptosis. A further study demonstrated that IL-6 is responsible for drug resistance and anti-apoptotic effects in prostatic cancer cells. The anti-apoptotic ability of IL-6 is associated with expression of the Bcl-2 family proteins [82]. Interestingly, the IL-6 mediated inhibition of apoptosis is associated with up-regulation of Bcl-2, which is independent of STAT3 [83]. We found increased IL-6 levels after doxorubicin treatment. However there were increased levels of STAT3 in combination with decreased levels of Bcl-2 at the same time. In conclusion the pathway-analyses offer doxorubicin mediated apoptotic and anti-apoptotic mechanisms side by side.

The concept that anticancer therapies primarily act by triggering apoptosis has been challenged, since a consistent link between the ability of tumor cells to undergo apoptosis in vitro and their susceptibility to anticancer therapy in vivo is not essential. Therefore, pathways leading to non-apoptotic cell death, e.g. necrosis or yet unclassified forms of cell death, may mediate the response to cytotoxic therapy [84,85]. Also, caspase independent apoptosis has been found to be induced by anticancer drugs in some cells [86-88]. Furthermore, recent findings have shown that caspase inhibitors do not protect cancer cells from death by cytotoxic agents, but may switch drug-induced apoptosis to an alternative default death e.g. sensescence [89].

Understanding these events and the diverse modes of tumor cell death may provide new opportunities for pathway-based rational therapy and for drug development [90].

The drug response is of course dependent on their concentration. Further studies including different sarcoma cells, drug concentrations and time responses will strengthened the knowledge about the chemotherapy resistance in the very heterogeneous soft tissue sarcomas.

## Conclusion

The analysis of gene expression patterns and regulated pathways, which result from treatment with cytostatic agents, could provide a new basis for tumor therapy. This study demonstrates that HT1080-fibrosarcoma cells reveal a specific response pattern of apoptosis-related factors on mRNA level, which depends on the used cytostatic drug. The response rates on the gene expression level, i.e. the number of genes regulated by the drugs, correlate to the clinical effectiveness of the drugs. In addition we found a large number of differentially expressed genes whose biochemical functions include cell proliferation, cell cycle regulators, cell growth, metabolism and others. These findings provide an insight into the complex biological processes affected by the drug and may represent the signature profiles of drug induced gene expression. The knowledge of the genes affected by the drugs in HT1080 fibrosarcoma cells will help to understand the diverse modes of cell death in other soft tissue sarcomas in response to cytotoxic therapies.

## Competing interests

The author(s) declare that they have no competing interests

## Authors' contributions

ML: coordinated the work, developed the study design and prepared the manuscriptLKH: carried out statistical analyses, prepared the manuscript and figures, have given substantial contribution to conception and designCK: prepared the manuscript and figures

HH: carried out cell culture, RNA isolation, microarray and PCR

AD: participated in the study design, prepared the figures and was helpful in preparing the manuscript

HUS: developed the idea, have given final approval of the version to be published

LS: carried out RNA isolation, microarray and PCR

SR: carried out the microarrays

LS: carried out RNA isolation and PCR

OM: developed the idea, study design and conceived the work

## Pre-publication history

The pre-publication history for this paper can be accessed here:



## Supplementary Material

Additional File 1 Validation of 46 selected candidate genes by RT-PCR signal log ratio values of 46 selected genes. Microarray and PCR data are displayed for comparison Click here for file

## Figures and Tables

**Table 1:** Hierarchical filter analysis for selection of substantially altered expression levels

Filter 1	change: increased – decreased
Filter 1	change: increased – decreased
Filter 3	SLR*<1.32 and Change p-value >0.999 and at least one Present (P) Detection call in control or treated array
	SLR*>1.32 and Change p-value <0.001 and at least one Present (P) Detection call in control or treated array

* SLR: Signal log Ratio

**Table 2:** Distribution of the probesets affected by the various drugs to categories of the GO biological categories.

	actinomycin D	doxorubicin	vincristine	HG-U133A
Total number of probesets in target list	3309	1019	134	22215
Total number of probesets with annotated biological process	2158	702	87	13704
				
Physiological process				
Cell growth and/or maintenance	827***	262*	40*	4701
Cell proliferation	367***	123***	14	1576
Cell cycle	290***	87***	10	1085
Death	151***	39	5	682
Metabolism	1507***	429	50	8247
Regulation of biological process				
Regulation of programmed cell death	87***	22	2	400
Anti-apoptosis	41***	5	2	152
Positive regulation of apoptosis	50*	10	0	231
Regulation of signal transduction	49*	13	3	242
I-κB kinase/NF-κB cascade	29*	7	2	123
Regulation of metabolism	509***	121	22*	2398
Protein biosynthesis	54***	6	1	156
Nucleobase, nucleoside, nucleotide synthesis	455***	111	21*	2232
Negative regulation of nucleobase, nucleoside, nucleotide and nucleid acid metabolism	49***	8	2	180
DNA metabolism	15**	1	0	50

Based on the 'Gene Ontology Standard Vocabulary' the probesets affected by actinomycin D, doxorubicin and vincristine were sorted according to the 'Biological Process' into the indicated major categories. The number of probesets with annotated biological processes is given and corresponds to approximately 66% of the probesets affected. The two major categories investigated are shown in bold and subcategories of the previous process are indicated by offset. As a reference the distribution of all annotated probesets of the HG-U133A array to the same categories are listed. Genes significantly overrepresented in the target lists were identified using the statistics (hypergeometric distribution) implemented in the 'GO browser' of Spotfire DecisionSite for Functional Genomics. *, ** and *** indicate p-values of p < 0.05, p < 0.01, and p < 0.001, respectively.
