# Supplementary material for: Response rate of fibrosarcoma cells to cytotoxic drugs on the expression level correlates to the therapeutic response rate of fibrosarcomas and is mediated by regulation of apoptotic pathways
Source: BMC Cancer. 2005 Jul 7;5:74. doi: 10.1186/1471-2407-5-74 (PMC1183194; doi:10.1186/1471-2407-5-74)
Supplement: Additional File 1 — Validation of 46 selected candidate genes by RT-PCR signal log ratio values of 46 selected genes. Microarray and PCR data are displayed for comparison [file 1471-2407-5-74-S1.doc]

|  | **Affy-ID** | **Assay-ID** | **Gene symbol** | **Doxo. 24h PCR SLR** | **Vinc. 24h PCR SLR** | **Actino. 24h PCR SLR** | **Doxo. 24h vs Co SLR** | **Doxo. 24h vs Co Change** | **Vinc. vs Co SLR** | **Vinc. vs Co Change** | **Actino. Vs Co SLR** | **Actino. Vs Co Change** |
| --- | --- | --- | --- | --- | --- | --- | --- | --- | --- | --- | --- | --- |
| 1 | 200887_s_at | Hs00234829_m1 | STAT1 | 2,341 | 1,042 | -0,691 | 1,64 | I | 0,54 | I | -0,64 | I |
| 2 | 202431_s_at | Hs00153408_m1 | MYC | -1,487 | -0,15 | -2,316 | -1,82 | D | -0,5 | D | -2,78 | D |
| 3 | 202535_at | Hs00356603_g1 | FADD | 0,907 | 1,043 | -2,545 | 0,37 | I | 0,59 | NC | -4,99 | D |
| 4 | 203265_s_at | Hs00387426_m1 | MAP2K4 | 1,45 | 0,743 | -2,586 | 0,99 | I | 0,29 | NC | -2,59 | D |
| 5 | 203276_at | Hs00194369_m1 | LMNB1 | -1,855 | 0,488 | -3,535 | -1,76 | D | -0,23 | NC | -3,98 | D |
| 6 | 203845_at | Hs00187332_m1 | PCAF | 3,348 | 1,853 | -0,322 | 2,1 | I | 0,62 | I | -1,15 | NC |
| 7 | 204859_s_at | Hs00559421_m1 | APAF1 | 4,161 | 1,114 | -3,448 | 3,24 | I | 0,26 | NC | -2,29 | MD |
| 8 | 205207_at | Hs00174131_m1 | IL6 | 2,467 | 1,685 | -3,301 | 1,76 | I | 1,28 | I | -1,06 | NC |
| 9 | 208328_s_at | Hs00271535_m1 | MEF2A | -1,26 | -1,5 | -2,932 | 1,51 | I | 0,58 | NC | -2,1 | NC |
| 10 | 208992_s_at | Hs00374280_m1 | STAT3 | 2,468 | 0,845 | -0,837 | 2,06 | I | 0,41 | NC | -0,52 | NC |
| 11 | 209790_s_at | Hs00154250_m1 | CASP6 | 2,993 | 1,359 | -2,816 | 2,26 | I | 1,06 | I | -1,73 | NC |
| 12 | 211537_x_at | Hs00177373_m1 | MAP3K7 | 1,103 | 0,581 | -3,123 | 0,28 | NC | -0,06 | NC | -3,32 | D |
| 13 | 212501_at | Hs00270923_s1 | CEBPB | 2,067 | 2,011 | -2,649 | 1,37 | I | 1,04 | I | -3,26 | D |
| 14 | 212983_at | Hs00610483_m1 | HRAS | 3,16 | 1,241 | -1,389 | 2,85 | I | 0,87 | I | -1,45 | NC |
| 15 | 213373_s_at | Hs00154256_m1 | CASP8 | 2,771 | 1,625 | -4,808 | 2,94 | I | 0,54 | I | -2,68 | D |
| 16 | 217373_x_at | Hs00234753_m1 | MDM2 | 0,152 | 0,299 | -3,271 | -0,05 | NC | -1,93 | D | -0,71 | NC |
| 17 | AFFX-HUMGAPDH/M33197_M_at | 4342376 | GAPDH | 0 | 0 | 0 | -0,14 | NC | -0,05 | NC | -0,43 | I |
| 18 | 204450_x_at | Hs00163641_m1 | APOA1 | non informative PCR |  |  | -2,6 | NC | -1,19 | NC | -0,71 | NC |
| 19 | 209364_at | Hs00188930_m1 | BAD | 0,786 | 1,28 | -1,819 | 1,01 | NC | 0,88 | NC | -0,62 | NC |
| 20 | 208478_s_at | Hs00180269_m1 | BAX | -0,163 | 0,057 | -1,952 | -0,36 | D | -0,62 | D | -1,7 | NC |
| 21 | 207004_at | Hs00608023_m1 | BCL2 | -0,044 | 2,66 | -4,349 | -0,48 | NC | -0,38 | NC | -1,1 | NC |
| 22 | 206011_at | Hs00354836_m1 | CASP1 | 3,835 | 1,648 | -1,668 | 1,44 | I | -0,08 | NC | -1,01 | NC |
| 23 | 211888_x_at | Hs00154268_m1 | CASP10 | 3,479 | 0,743 | -0,008 | 0,83 | I | 0,41 | NC | -2,1 | NC |
| 24 | 208050_s_at | Hs00154242_m1 | CASP2 | 0,78 | 1,403 | -2,416 | 0,64 | NC | 0,32 | NC | -1,07 | NC |
| 25 | 202763_at | Hs00234387_m1 | CASP3 | 2,579 | -0,129 | -3,465 | 2,28 | I | -0,27 | NC | -2,48 | D |
| 26 | 213596_at | Hs00426677_m1 | CASP4 | -0,308 | 0,889 | -1,534 | 0,43 | NC | 0,22 | NC | -1,51 | NC |
| 27 | 207500_at | Hs00237061_m1 | CASP5 | non informative PCR |  |  | 0,38 | NC | 0,54 | NC | -0,58 | NC |
| 28 | 207181_s_at | Hs00169152_m1 | CASP7 | 1,627 | 1,17 | -3,52 | 0,8 | I | 0,06 | NC | -3,56 | D |
| 29 | 210775_x_at | Hs00154260_m1 | CASP9 | non informative PCR |  |  | 2,35 | I | 0,07 | NC | -1,5 | NC |
| 30 | 209616_s_at | Hs00275607_m1 | CES1 | non informative PCR |  |  | 0,17 | NC | -1,11 | NC | -2,94 | NC |
| 31 | 201066_at | Hs00357717_m1 | CYC1 | 0,986 | 0,309 | -1,052 | 0,97 | I | -0,14 | NC | -1,55 | NC |
| 32 | 2028_s_at | Hs00153451_m1 | E2F1 | 0,104 | 1,15 | -1,509 | -0,31 | NC | 0,05 | NC | -1,14 | NC |
| 33 | 210984_x_at | Hs00193306_m1 | EGFR | 0,484 | 1,165 | -3,03 | -1,06 | NC | -2,91 | NC | -1,5 | NC |
| 34 | 203619_s_at | Hs00202349_m1 | FAIM2 | non informative PCR |  |  | -0,08 | NC | -0,44 | NC | -1,4 | NC |
| 35 | 215913_s_at | Hs00169604_m1 | GULP1 | non informative PCR |  |  | -0,68 | D | 0,33 | NC | -0,93 | NC |
| 36 | 207062_at | Hs00169095_m1 | IAPP | non informative PCR |  |  | 1,39 | NC | 0,16 | NC | -0,26 | NC |
| 37 | 208441_at | Hs00181385_m1 | IGF1R | 2,028 | 0,947 | -2,365 | -0,9 | NC | -0,28 | NC | -0,94 | NC |
| 38 | 203411_s_at | Hs00153462_m1 | LMNA | 0,981 | 0,014 | -1,163 | 0,52 | NC | -0,25 | NC | -1,39 | NC |
| 39 | 208351_s_at | Hs00177066_m1 | MAPK1 | 1,003 | 0,941 | -1,189 | 0,48 | I | 0,01 | NC | -1,29 | NC |
| 40 | 209239_at | Hs00765730_m1 | NFKB1 | 1,535 | 1,538 | -3,811 | 0,71 | I | 0,57 | NC | -1,83 | D |
| 41 | 205170_at | Hs00237139_m1 | STAT2 | 2,412 | 1,365 | -0,616 | 1,53 | I | 0,67 | NC | -0,85 | NC |
| 42 | 206605_at | Hs00195731_m1 | P11 | non informative PCR |  |  | 0,26 | NC | -0,1 | NC | -0,97 | NC |
| 43 | 204201_s_at | Hs00196632_m1 | PTPN13 | 1,598 | 0,449 | -3,751 | 0,72 | I | -0,07 | NC | -2,07 | NC |
| 44 | 207569_at | Hs00177228_m1 | ROS1 | 1,117 | 2,671 | -2,504 | -0,21 | NC | -0,38 | NC | -0,33 | I |
| 45 | 212780_at | Hs00362308_m1 | SOS1 | 1,234 | 1,218 | -3,19 | 0,51 | I | 0,46 | I | -2,71 | D |
| 46 | 207113_s_at | Hs00174128_m1 | TNF | -1,967 | 1,5 | 2,922 | 2,32 | NC | 1,94 | NC | 1,84 | NC |
| 47 | 211300_s_at | Hs00153349_m1 | TP53 | 1,352 | 0,122 | -0,659 | 0,65 | I | -0,13 | NC | -1,07 | NC |

SLR: Signal Log Ratio

I: increased

D: decreased

NC: not changed

Con: Control (untreated HT1080 cells)
